# The impact of cathodal tDCS on the GABAergic system in the epileptogenic zone: A multimodal imaging study

**DOI:** 10.3389/fneur.2022.935029

**Published:** 2022-08-05

**Authors:** Sulaiman I. Abuhaiba, Isabel C. Duarte, João Castelhano, Ana Dionísio, Francisco Sales, Richard Edden, Miguel Castelo-Branco

**Affiliations:** ^1^Coimbra Institute for Biomedical Imaging and Translational Research (CIBIT), University of Coimbra, Coimbra, Portugal; ^2^Institute of Nuclear Sciences Applied to Health (ICNAS), University of Coimbra, Coimbra, Portugal; ^3^Epilepsy Unit, Faculty of Medicine, Clinical and Academic Center (CCAC), Coimbra, Portugal; ^4^Faculty of Medicine, Clinical and Academic Center (CCAC), University of Coimbra, Coimbra, Portugal; ^5^Department of Radiology, The Johns Hopkins University School of Medicine, Baltimore, MD, United States; ^6^FM Kirby Center for Functional MRI, Kennedy Krieger Institute, Baltimore, MD, United States

**Keywords:** GABA, pharmacoresistant epilepsy, tDCS, MRS, epileptiform interictal discharges

## Abstract

**Objectives:**

We aimed to investigate the antiepileptic effects of cathodal transcranial direct current stimulation (c-tDCS) and mechanisms of action based on its effects on the neurotransmitters responsible for the abnormal synchrony patterns seen in pharmacoresistant epilepsy. This is the first study to test the impact of neurostimulation on epileptiform interictal discharges (IEDs) and to measure brain metabolites in the epileptogenic zone (EZ) and control regions simultaneously in patients with pharmacoresistant epilepsy.

**Methods:**

This is a hypothesis-driven pilot prospective single-blinded repeated measure design study in patients diagnosed with pharmacoresistant epilepsy of temporal lobe onset. We included seven patients who underwent two sessions of c-tDCS (sham followed by real). The real tDCS session was 20 min in duration and had a current intensity of 1.5 mA delivered *via* two surface electrodes that had dimensions of 3 × 4 cm. The cathode electrode was placed at FT7 in the center whereas the anode at Oz in the center. After each session, we performed electroencephalographic recording to count epileptiform IEDs over 30 min. We also performed magnetic resonance spectroscopy (MRS) to measure brain metabolite concentrations in the two areas of interest (EZ and occipital region), namely, gamma-aminobutyric acid (GABA), glutamate (Glx), and glutathione. We focused on a homogenous sample where the EZ and antiepileptic medications are shared among patients.

**Results:**

Real tDCS decreased the number of epileptiform IEDs per min (from 9.46 ± 2.68 after sham tDCS to 5.37 ± 3.38 after real tDCS), *p* = 0.018, as compared to sham tDCS. GABA was decreased in the EZ after real c-tDCS stimulation as compared to sham tDCS (from 0.129 ± 0.019 to 0.096 ± 0.018, *p* = 0.02). The reduction in EZ GABA correlated with the reduction in the frequency of epileptiform IED per min (rho: 0.9, *p* = 0.003).

**Conclusion:**

These results provide a window into the antiepileptic mechanisms of action of tDCS, based on local and remote changes in GABA and neural oscillatory patterning responsible for the generation of interictal epileptiform discharges.

## Introduction

Epilepsy is a very common neurologic condition that has a prevalence of around 1% and a yearly incidence of 61/100,000 (1). A significant proportion (up to 40%) of patients remain intractable and are diagnosed with pharmacoresistant epilepsy (2). Surgical intervention remains the mainstay of treatment in those patients with focal onset; however, this may be associated with significant neurologic complications (3). Moreover, symptomatic freedom after resection surgery is estimated to be around 70% (3). Because of these, novel translational science approaches leading to less invasive treatments of drug-resistant epilepsy are needed. One of such possible new treatments is the utilization of noninvasive brain stimulation.

Epilepsy has historically been seen as a disorder of hypersynchronization/desynchronization. This places gamma-aminobutyric acid (GABA), the main inhibitory neurotransmitter, as a putative main mechanistic player. GABAergic inhibition enables synchronization of activity in neuronal networks and underlies oscillations related to normal cognitive functions (4–6). However, GABAergic inhibition might be responsible for the abnormal synchronization that leads to the generation of pathologic high-frequency oscillations, seen in patients with epilepsy, especially temporal lobe epilepsy (TLE) (6). GABA, the principal inhibitory neurotransmitter in the brain, has long been thought to be related to epileptogenesis (7–9). Galanopoulou showed that immature GABAergic inhibitory systems may contribute to epileptogenesis (10). For instance, Cepeda et al. (11) and Cherubini et al. (12) show that predominant GABAergic synaptic transmission in an immature neuronal network may lead to depolarization and excessive cell firing, where GABA acts as an excitatory neurotransmitter. Patients with drug-resistant epilepsy are reported to have a higher frequency of spontaneous inhibitory post-synaptic currents, which are related to the generation of pathologic high-frequency oscillations (13). Moreover, there is current evidence of increased GABA receptor activity relative to glutamate (Glx) receptor activity in severe cases of cortical dysplasia (14). Focal cortical dysplasia and tuberous sclerosis are possible etiologies of drug-resistant epilepsy. It has been reported that GABA concentration is found to be markedly increased in the epileptogenic zone (EZ) in seizure disorders that are characterized by an abnormal cortex (15). There is also an almost consensus now on increased levels of GABA in *ex vivo* spectroscopy experiments of brain biopsies from patients with drug-resistant epilepsy (16–18).

Seizures are the result of hypersynchronous neuronal discharges, which result in the summation of the action potentials of multiple neurons in the EZ, at least in the initiation phase. The summation of the post-synaptic currents results in large amplitude epileptic electroencephalographic patterns. GABAergic inhibition is expected to play a critical role in the generation of this pathologic hypersynchronous state in the EZ (19). *In vitro* studies show that gamma frequency oscillations, which are dependent on GABAergic synchronization, are responsible for physiologic gamma oscillations and also pathologic oscillations seen in epilepsy (19). However, in the case of epilepsy, the level of local and neuronal spike synchrony in the EZ is much higher than in normal tissues (20–22).

Synchrony in the context of epilepsy represents quite a complex matter. While there is evidence of local and neuronal spike hypersynchronization, there is also strong evidence of desynchrony at a larger scale (23).

A noninvasive stimulation method that has some form of antiepileptic effect should in theory modulate the neurotransmitters responsible for these abnormal and complex synchrony patterns seen in epilepsy or modulate synapses. Transcranial direct current stimulation (tDCS) is a safe method of brain stimulation (24).

Fregni et al. (25) showed that cathodal tDCS (c-tDCS) stimulation of the EZ in patients with drug-resistant epilepsy decreased cortical excitability in the epileptogenic focus. Auvichayapat et al. (26) also studied the antiepileptic efficacy of c-tDCS in 36 children with drug-resistant epilepsy. C-tDCS was efficient in decreasing epileptiform discharges for 48 h. Yook et al. (27) reported dramatic results when they used c-tDCS on an 11-year-old girl with drug-resistant epilepsy due to focal cortical dysplasia. The antiepileptic effects of c-tDCS are expected to come into play by decreasing cortical excitability and altering synaptic efficacy (28). c-tDCS is shown to decrease the number of epileptic spikes, the firing rate of pyramidal cells, and suppressed GABAergic interneurons in a simulation model (29). In this explicit model, they used an epileptogenic network that is capable of producing spike-like events involving more GABAergic neurons, which is in agreement with other experimental studies (30). However, the exact mechanism of c-tDCS inhibitory effects in epilepsy is still unknown (31). In this study, we aimed to investigate the mechanistic role of c-tDCS in terms of inhibition/excitation balance and epileptogenic brain activity assessed as the frequency of epileptiform interictal discharges (IEDs) per min and to shed light on its role as an antiepileptic. We tested the hypothesis that c-tDCS modulates GABAergic inhibition and its mediated local synchrony in the EZ as compared to a reference area in the occipital region. A multimodal imaging approach with magnetic resonance spectroscopy (MRS) and EEG was used to measure the effects of c-tDCS on brain metabolites and cortical epileptogenesis respectively after sham and real tDCS. Changes in metabolites are measured by MRS, whereas neurophysiologic effects are assessed with electroencephalography (EEG). Our hypothesis was that c-tDCS would decrease cortical excitability and this could be measured by a reduction in the number of epileptiform discharges in the EEG. To our knowledge, this is the first study to test for the impact of neurostimulation on pathological epileptiform IED and to measure GABA, Glx, and glutathione in the EZ and occipital area simultaneously after real c-tDCS in patients with pharmacoresistant TLE.

## Methods

### Participants

This is a hypothesis-driven pilot prospective single-blinded repeated measure design study in patients diagnosed with pharmacoresistant epilepsy of temporal lobe onset. Seven participants with pharmacoresistant epilepsy were included. Inclusion criteria were the following: age above 18 years, diagnosis of pharmacoresistant epilepsy as defined by the International League Against Epilepsy, and nonpregnant and nonlactating in women. Exclusion criteria were skin conditions, such as eczema, the metal inside the head but outside the mouth, implanted devices, such as cardiac pacemaker, cochlear implant, or vagal nerve stimulation device, history of recurrent or severe headaches, and presence of other comorbid neurologic conditions. All procedures used in this study conformed to the Declaration of Helsinki, and the protocol was approved by the Faculty of Medicine of the University of Coimbra Research Ethics Committee. Written informed consent was obtained from each participant and they accepted that their pieces of clinical information were to be used in the scope of this research project and for any publications that may result from this work.

Participants were recruited from the epilepsy monitoring unit at Centro Hospitalar e Universitário de Coimbra. The small number of patients is due to the following reasons: (1) we wanted to include patients with left-onset temporal seizures to uniform the stimulation protocol, (2) we included patients who in their previous 24-h EEG had a large number of IED during awake and sleep states, and (3) patients should have not been seizure-free in the last 3 months.

Participants were contacted 2 weeks after their discharge from the epilepsy monitoring unit and invited to participate in the study, which was conducted at the Institute of Nuclear Sciences Applied to Health (ICNAS), University of Coimbra, Portugal. Patients were instructed to not make any changes to their medication while included in the study.

Participants visited ICNAS two times. The first visit was to explain the experiments and to perform sham tDCS. The participants did not know that they will be starting with sham tDCS. One week later, the participants came for a second visit to ICNAS to perform real tDCS. After tDCS in each visit, EEG and MRS were performed. Table 1 summarizes the demographic data of the included patients and their clinical information.

**Table 1:** Clinical and demographic description of the included subjects.

**ID**	**IED per minute^+^**	Localization^*^	**Age**	**Age at onset**	**Medications**	**Etiology^&^**
1	5.5	Left frontotemporal	20	8	Levetiracetam	Hippocampal sclerosis
					Valproate	
					Perampanel	
2	15.0	Left temporal	32	22	Levetiracetam	FCD
					Valproate	
3	10.0	Left temporal	21	14	Valproate	Hippocampal sclerosis
					Perampanel	
4	13.2	Left temporal	33	22	Levetiracetam	Hemartoma
					Valproate	
5	9.6	Left temporal	35	17	Levetiracetam	Tuberus
					Valproate	
6	5.6	Left temporal	46	40	Levetiracetam	FCD—neocortical
					Valproate	
7	7.3	Left temporal	37	23	Levetiracetam	Suspected FCD
					Valproate	

+ *During 30 min of acquisition*.

* *Localization is based on the multidisciplinary team consensus based on multimodal imaging, interictal, ictal EEG, and ictal semiology*.

*FCD, focal cortical dysplasia*.

& *Based on epilepsy protocol structural MRI as interpreted by a neuroradiologist*.

### Lobar localization of the presumed EZ

The presumed EZ was determined based on a multimodal imaging approach utilizing MRI, fluorodeoxyglucose (FDG)-PET, and ictal-single-photon emission computerized tomography (SPECT) when available in addition to scalp EEG. A multidisciplinary meeting was held to discuss each participant before their enrolment in the study as part of their presurgical evaluation. Based on imaging findings and clinical semiology, lobar localization was determined in the multidisciplinary meeting.

### Transcranial direct current stimulation

All participants underwent sham tDCS in the first visit followed by real tDCS. tDCS was performed in a quiet room at ICNAS using a Soterix Medical 1 × 1 tDCS Low-Intensity Stimulator (Soterix, New York, USA). Participants were blinded to the nature of the intervention.

Participants underwent a 20 min stimulation session at 1.5 mA continuous current delivered to the brain *via* two surface electrodes put in between saline-soaked sponges. Both electrodes had the same dimensions of 3 × 4 cm. The cathode electrode was placed at FT7 in the center and the anode at Oz in the center.

The position of the tDCS electrodes was chosen based on the results from COMETS: a MATLAB toolbox that can stimulate local electric fields generated by tDCS (32). First, a previous anatomical 3D MRI (MPRAGE sequence) was used to extract the main three boundaries (brain, bone, and scalp skin using Curry7), which were needed for the COMETS toolbox to generate an individualized head model for each patient. Then, the positions of the electrodes were chosen manually on the scalp surface at FT7 and Oz at the center of the cathode and anode electrodes, respectively. Third, the tDCS parameters were entered in the COMETS toolbox (current intensity of 1.5 mA and sponge size of 3 × 4 cm). Finally, the COMETS toolbox was used to compute the current density maps, which showed that the target zones (EZ and Occipital regions) were to be reached by the electric current field (see Supplementary Figure S2).

### Electroencephalography

Scalp electroencephalography was performed immediately before and after each tDCS session (sham or real). EEG was used to count the number of IEDs, which was done by an expert epileptologist. The duration of each EEG acquisition was 30 min.

Electroencephalography was recorded using a 64 electrodes cap (QuickCap, NeuroScan, USA) with the electrodes placed according to the extended 10/20 system. The electrode impedances were kept below 5 kΩ. The signal was amplified and recorded at a sampling rate of 1 kHz, low pass filter at 200 Hz, using a SynAmps2/RT amplifier (NeuroScan, USA).

An electroencephalographic signal was recorded using Scan 4 (NeuroScan, USA), with the acquisition reference electrode placed at a half distance between CZ and FCZ.

Data analysis was performed with Brainstorm (33), which is documented and freely available for download online under the GNU general public license (http://neuroimage.usc.edu/brainstorm). The EEG signal was down sampled to 400 Hz and band filtered between 1 and 100 Hz.

The dataset was cleaned using an automatic rejection tool with a threshold of 120 μV for all electrodes, and this was followed by a visual inspection to ensure the data were free from artifacts. Rejected channels due to abnormal noise activity were interpolated using spherical spline interpolation. The recordings were re-referenced to the average of all remaining channels.

### MRS acquisitions and analysis

Patients underwent anatomical and spectroscopy imaging using a Siemens 3T Scanner (Siemens Magnetom 3 T Tim Trio, Erlangen, Germany). T1-weighted structural images of the brain were acquired with an MPRAGE sequence with 1 mm^3^ isotropic voxel, repetition time 2.53 s, echo time 3.42 ms, inversion time 1,100 ms, flip angle 7°, field of view 256 × 256 mm^2^, 256 × 256 matrix, 176 slices, and GeneRalized Autocalibrating Partial Parallel Acquisition (GRAPPA) acceleration factor = 2.

**Figure 1:**
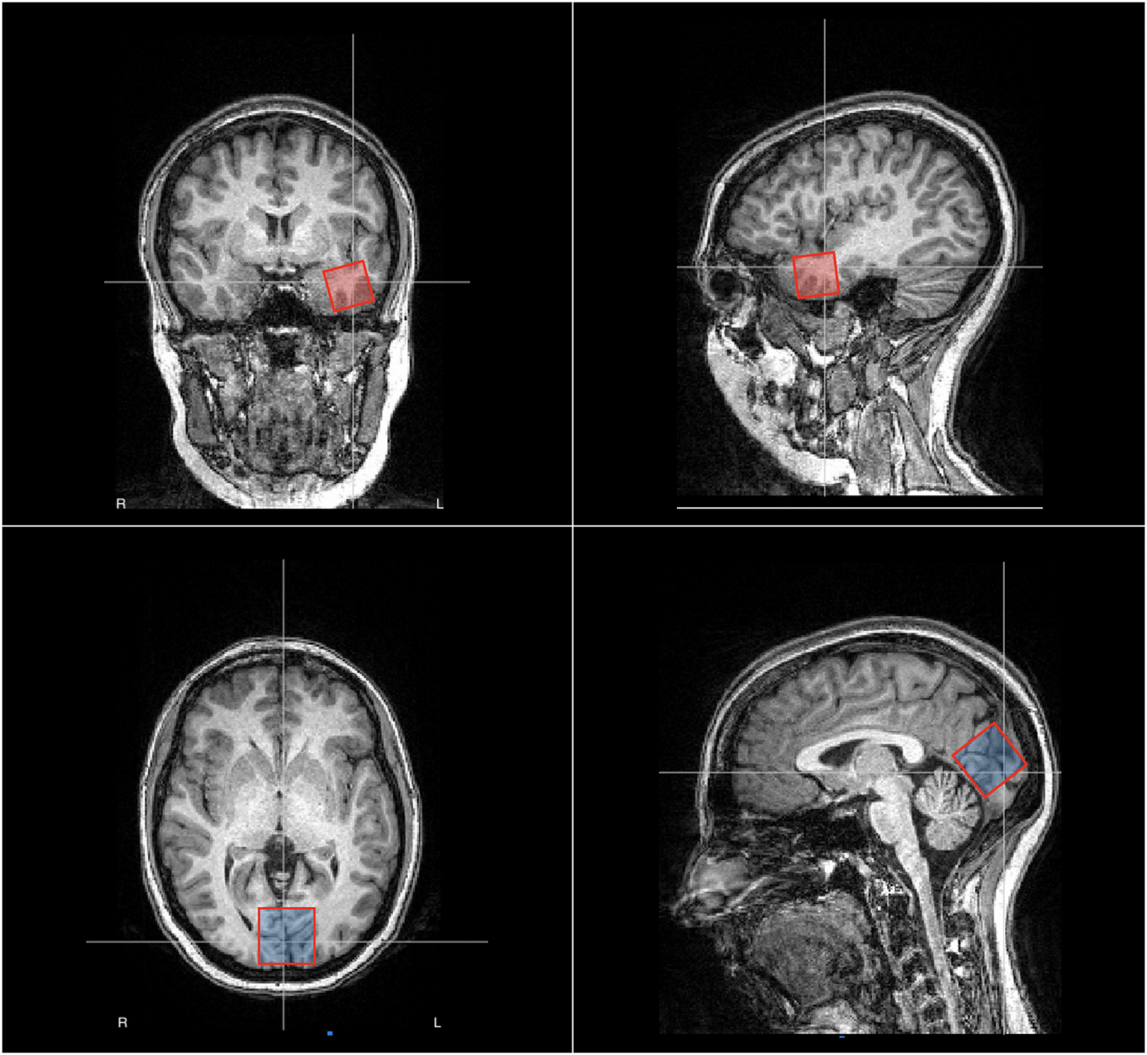
Placement of the voxels for magnetic resonance spectroscopy (MRS) in patient no. 2, male, age 32 years. In red, we show the EZ voxel, which is positioned in the left anterior temporal lobe whereas in blue we show the reference voxel, which is positioned in the occipital zone medially. The EZ voxel was measured 25 × 25 × 25 mm whereas the occipital voxel was measured 30 × 30 × 30 mm.

T1-weighted images were analyzed for structural abnormalities. Two MRS voxels were positioned one in the left anterior temporal lobe and another medially in the occipital cortex, as shown in Figure 1. The EZ voxel was measured 15.625 cm^3^ whereas the occipital voxel had a volume of 27 cm^3^. We opted for a smaller volume in the temporal region because of the presence of bone and cerebrospinal fluid (CSF) in the selected area, which rendered larger voxel volumes too noisy.

Data were acquired on a 3T Siemens Scanner (Siemens Magnetom 3T Tim Trio, Erlangen, Germany). T1-weighted structural images were acquired with an MPRAGE sequence with 1 mm^3^ isotropic voxel, repetition time 2.53 s, echo time 3.42 ms, inversion time 1,100 ms, flip angle 7, field of view 256 × 256 mm^2^, 256 × 256 matrix, 176 slices, and GRAPPA acceleration factor = 2. We used the Hadamard Encoding and Reconstruction of MEGA-Edited Spectroscopy (HERMES) approach (34) to quantify GABA, Glx, and glutathione. Two voxels were chosen for each patient, one in the left anterior temporal lobe and the second in the bilateral occipital lobe. The volume of the EZ voxel in the temporal lobe was 15.625 cm^3^ whereas the occipital voxel had a volume of 27 cm^3^. The structural images were analyzed for structural abnormalities and bright object detection. The HERMES data were processed with Gannet software (35). GABA, Glx, and glutathione in addition to creatine signals were obtained from the different edited spectra. The peaks for each metabolite were fitted to a simple Gaussian model. Creatine signal was fitted to a double Lorentzian model. Results were expressed as levels of metabolite/Cr. Normalization to creatine was used to reduce intersubject variance from both different signal-to-noise levels and CSF fraction within the voxel (36). GABA signal is known to be contaminated by other macromolecules (37), therefore, we refer to GABA from now on as GABA+. Model fit errors for all spectra were set at <10% to be accepted. Spectra with higher fit error for any of the metabolites were considered as missing values.

### Statistical analysis

Statistical analyses were performed with IBM SPSS Statistics version 20 software. Nonparametric tests for related samples (Wilcoxon) and correlation (spearman's rho) were used. Our statistical significance threshold was set at a *p* < 0.05.

## Results

### Effects of tDCS on cortical excitability of the EZ (presented as the frequency of interictal epileptiform discharges per minute)

**Figure 2:**
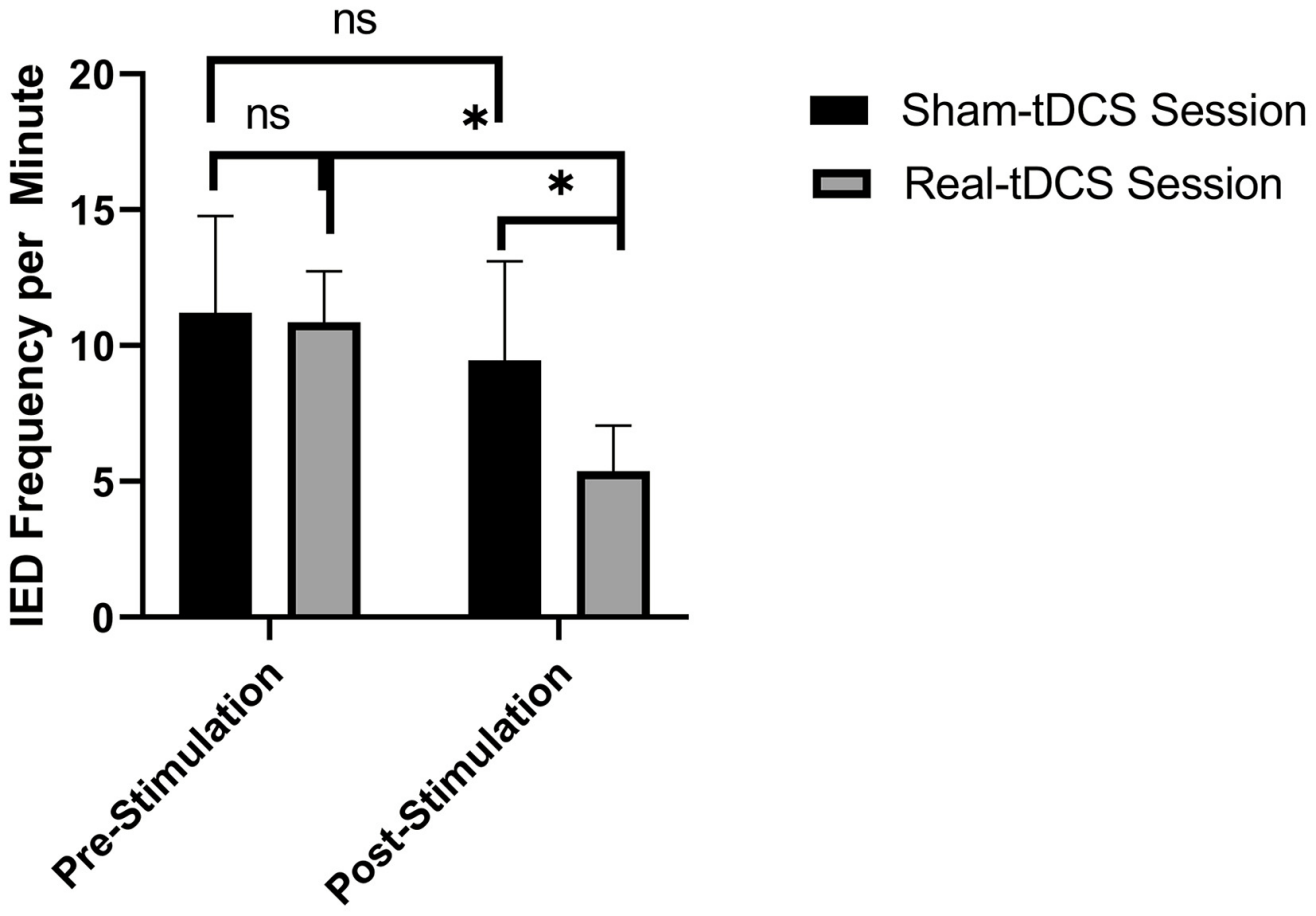
Real transcranial direct current (tDCS) decreased the interictal discharges (IEDs) frequency per minute from (10.86 ± 3.74) prestimulation to (5.37 ± 3.38) post-stimulation [*p* = 0.045] and from (9.46 ± 2.74) after sham tDCS to (5.37 ± 3.38) after real tDCS [*p* = 0.018]. n.s.: not statistically significant, i.e., *p* > 0.05. * means statistically significant with a *p* < 0.05.

Real tDCS decreased the frequency of IEDs per minute from (10.86 ± 3.74) prestimulation to (5.37 ± 3.38) post-stimulation and from (9.46 ± 2.74) after sham tDCS to (5.37 ± 3.38) after real tDCS (*p* < 0.05 in both comparisons, Figure 2). Sham tDCS did not change the frequency of IEDs per minute immediately before the stimulation as compared to after sham stimulation.

### Effects of tDCS on neurotransmitter profile in the EZ

Gamma-aminobutyric acid and GABA/Glx ratio were decreased in the EZ after real tDCS as compared to after sham tDCS. Glx and glutathione were increased in the EZ after real c-tDCS stimulation of the EZ as compared to sham tDCS. Table 2 summarizes the changes in brain metabolites in the EZ after sham- and real tDCS stimulation.

**Table 2:** Effects of transcranial direct current (tDCS) stimulation on brain metabolites.

	**Post sham-tDCS**	**Post real-tDCS**	**p-value**
**Changes in the Epileptogenic Zone:**			
**GABA**	**0.129** **+** **0.019**	**0.096** **+** **0.018**	**0.02**
Glx	0.08 ± 0.004	0.093 ± 0.003	NS
**Glutathione**	**0.031** **+** **0.007**	**0.048** **+** **0.004**	**0.02**
**GABA/Glx ratio**	**1.611** **+** **0.222**	**1.079** **+** **0.195**	**0.03**
N (subjects)	7	7	
**Changes in the Occipital Area:**			
**GABA**	**0.098** **+** **0.004**	**0.086** **±0.004**	**0.02**
Glx	0.087 + 0.003	0.088 + 0.002	NS
Glutathione	0.051 + 0.005	0.046 + 0.003	NS
**GABA/Glx ratio**	**1.129** **+** **0.053**	**0.994** **+** **0.081**	**0.04**
N (subjects)	7	7	

*The cathode was placed over the left temporal area whereas the anode was over the occipital area in the seven subjects. The Wilcoxon Signed-Rank test was used to check for differences. Statistical significance was defined as a p-value <0.05. Bold values signify statistical significance, i.e. p <0.05*.

Supplementary Figure S2 in supplementary data shows the MRS spectra from one patient after sham vs. real tDCS stimulation from the EZ.

The only reduction in EZ GABA was correlated with the observed reduction in IED frequency (Spearman's rho correlation coefficient 0.9, *p* = 0.003).

### Effects of tDCS on neurotransmitters profile in the occipital area

Gamma-aminobutyric acid and GABA/Glx ratio were decreased in the occipital area after anodal stimulation as shown in Table 2. Changes in occipital brain metabolites did not correlate with the observed changes in IED frequency.

## Discussion

In the present study, we investigated the mechanisms of action of c-tDCS in the treatment of pharmacoresistant epilepsy of temporal lobe origin. c-tDCS was effective in suppressing interictal epileptiform discharges by approximately 50% after a single session confirming that cathodal DC polarization indeed decreases cortical excitability in the EZ in patients with left TLE. We found that cathodal stimulation of the EZ decreased GABA concentration. This is the first study to report this effect in human subjects and to correlate GABA changes with changes in IEDs. These results provide the biological basis for the reported efficacy of a single session of c-tDCS immediately after the session.

After sham tDCS, we show that GABA levels were significantly higher in the EZ as compared to the reference area in the occipital region. One of the possible hypotheses for the development of pharmacoresistant epilepsy is reduced sensitivity to GABA_A_ receptors to agent binding to the benzodiazepine receptor site 1 and other changes in GABA_A_ receptors were reported in brain tissues resected from patients with pharmacoresistant TLE (38). In common etiologies of drug-resistant epilepsies, such as cortical tubers in tuberous sclerosis or in patients with focal cortical dysplasia, it has been previously shown that extracellular GABA is markedly increased in the EZ (15, 16, 18, 39). A recent study clearly showed that pathological high-frequency oscillations are associated with increased GABAergic synaptic activity in the epileptic focus, further emphasizing the role of GABAergic interneurons in the generation of pathological high frequency oscillations (HFOs) (40). The current evidence points toward the idea that pathologic HFOs are epileptogenic, rather than a mere consequence of epileptogenesis. Interestingly, pacemaker GABA activity is also associated with pathologic HFOs, further confirming that GABA is not purely an inhibitory neurotransmitter when it comes to the EZ (40). On the other hand, it has been reported that GABA levels were low in patients with drug-sensitive epilepsy syndromes and the use of antiepileptic in that subgroup of patients was associated with an increment in GABA, which was associated with response to treatment (39). Therefore, one can conclude that GABAergic dysfunction in epilepsy is multifaceted and in patients with drug-resistant epilepsy as in our cohort one expects to find an increased GABA in the EZ, which agrees with our findings.

Cathodal stimulation of the EZ resulted in a decrease in GABA concentration, which has relevant clinical significance. We hypothesize that this decrease in GABA could be due to improved transportation of GABA, increased degradation of GABA, or other unexplored mechanisms. Surprisingly, the decrease in GABA in our study was correlated with a decrease in cortical excitability in the EZ as evidenced by a decrease in the frequency of epileptic discharges post-real tDCS. A recent modeling study suggested that cathodal stimulation of the EZ in a drug-resistant epilepsy model would mainly suppress GABAergic interneurons, which were associated with less epileptic spiking (29). Cathodal stimulation has been previously reported to decrease GABA by approximately 11% in an animal model (41). In our study, GABA in the EZ was decreased by approximately 25%. Froc et al. also reported that cathodal stimulation decreases Glx significantly; however, this was not observed in our study. Bilateral stimulation with the cathode placed at M1 has been reported to result in a decrease in GABA concentration in the cathode-stimulated area (42).

Gamma-aminobutyric acid degradation is dependent on the tricarboxylic acid (TCA) cycle and glutathione in its reduced form is needed for proper trafficking of GABA in the TCA cycle. In this study, we showed that glutathione in its reduced form is decreased in the EZ as compared to the reference area. Glutathione has been previously reported to be decreased in the EZ in patients with drug-resistant epilepsy measured by *in vivo* (1)H-MRS (43). Moreover, c-tDCS stimulation of the EZ has increased the concentration of glutathione, which is a potent antioxidant in the brain. This could be the explanation for the improved degradation of GABA and could explain why GABA is decreased in the EZ after tDCS. However, changes in glutathione concentration in the EZ did not correlate with the observed changes in the frequency of IED.

These findings suggest that a decrease in GABA in the EZ is associated with a decrease in epileptogenicity as measured by the number of IEDs per minute in patients with pharmacoresistant TLE (28).

Cathodal stimulation of the M1 region resulted in a decrease in GABA contralaterally (in relation to the cathode position) in a unilateral configuration and ipsilaterally in a bilateral configuration (under the cathode) as suggested by a previous study (42). The aftereffects of c-tDCS have been previously suggested to be dependent on the modulation of glutamatergic synapsis (28) with a significant decrease in Glx concentration within the stimulated cortex (44), however, we did not see this effect on Glx in our study. On the other hand, a decrease in GABA concentration within the stimulated cortex after c-tDCS has been reported before (44).

The post-stimulation effects of anodal tDCS are likely to depend on synaptic modulation. Transcranial magnetic stimulation (TMS) studies showed a decrease in GABAergic interneuronal activity locally under the anode post-stimulation (45). This is in agreement with an MRS study that showed a decrease in GABA concentration within the stimulated cortex by the anode electrode 10 min after anodal stimulation (44). This is in agreement with our finding of a decrease in occipital GABA after anodal stimulation. However, none of the changes in brain metabolites in the occipital area correlated with IEDs frequency reduction in our study. On the other hand, anodal stimulation could have a widespread effect on intracortical interneurons (GABAergic and Glutamatergic interneurons) (45). This could explain why in our nonunilateral configuration of tDCS electrodes (where both the anode and cathode target different brain regions in the patient), the area under the cathode did not have a significant change in the concentration of Glx.

In conclusion, our findings suggest that c-tDCS provides an antiepileptic effect in patients with pharmacoresistant epilepsy as it has been associated with a significant decrease in the frequency of interictal epileptiform discharges. The possible mechanism of action of c-tDCS according to our findings is the modulation of the GABAergic inhibition mediated synchrony in the EZ, with a reduction of GABAergic inhibition in the cathodal-stimulated region.

## Data availability statement

The raw data supporting the conclusions of this article will be made available by the authors, without undue reservation.

## Ethics statement

The studies involving human participants were reviewed and approved by Comissão de Etica da Faculdade de Medicina da Universidade de Coimbra. The patients/participants provided their written informed consent to participate in this study.

## Author contributions

SA has designed the experiments, recruited participants, performed the different tasks, analyzed the data, marked epileptiform interictal discharges, and wrote this manuscript. ID helped in designing the experiments and participant recruitment. JC helped with designing of the experiments and reviewed the manuscript. AD helped with patient recruitment and obtained the informed consent in Portuguese. FS reviewed the marking of epileptiform interictal discharges and reviewed the manuscript. RE reviewed the manuscript and provided advice. MC-B designed the experiments, reviewed the data analyses, helped in writing this manuscript, and supervised the work of SA. All authors contributed to the article and approved the submitted version.

## Funding

This work was supported by Fundação Luso-Americana para o Desenvolvimento [Prémio FLAD Life Sciences 2020] and Portuguese Foundation for Science and Technology (FCT), COMPETE, DSAIPA/DS/0041/2020, FCT-UID/04950 B&P/2020, BIGDATIMAGE, CENTRO-01-0145-FEDER-000016 financed by Centro 2020 FEDER, COMPETE, PAC—MEDPERSYST POCI-01-0145- FEDER-016428, and AIMS-2-Trials. This work applies tools developed under NIH R01 EB016089, R01 EB023963, and P41 EB031771. The funders had no role in study design, data collection and analysis, decision to publish, or preparation of the manuscript.

## Conflict of interest

The authors declare that the research was conducted in the absence of any commercial or financial relationships that could be construed as a potential conflict of interest.

## Publisher's note

All claims expressed in this article are solely those of the authors and do not necessarily represent those of their affiliated organizations, or those of the publisher, the editors and the reviewers. Any product that may be evaluated in this article, or claim that may be made by its manufacturer, is not guaranteed or endorsed by the publisher.
