# Supplementary material for: The impact of cathodal tDCS on the GABAergic system in the epileptogenic zone: A multimodal imaging study
Source: Front Neurol. 2022 Aug 5;13:935029. doi: 10.3389/fneur.2022.935029 (PMC9388822; doi:10.3389/fneur.2022.935029)
Supplement: Supplementary file 1 [file Data_Sheet_1.PDF]

Supplementary Figures:

Figure S1: A modeled current intensity map computed by the MATLAB toolbox COMETS which shows that the electric current supplied by the tDCS electrodes to be reaching the target regions (EZ under the cathode – electrode 1) and (OCC under the anode – electrode 2).

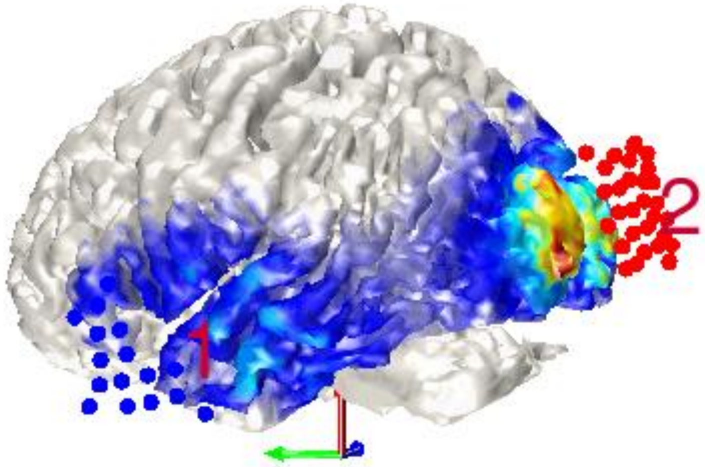

Figure S2: An example of the extracted MRS spectra using Gannet software and HERMES approach for GABA, Glx, and GSH from the epileptogenic zone after sham tDCS versus real tDCS stimulation. These spectra are from the same patient depicted in Figure 1.

<table>
<tr><td>After sham tDCS</td><td>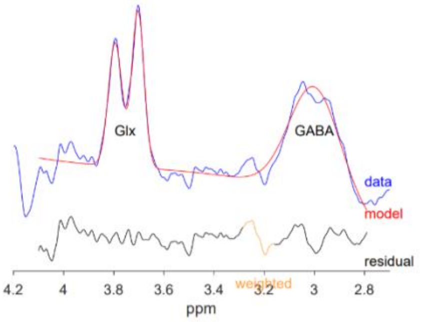
GABA/Cr: 0.12
Glx/Cr: 0.07
GABA/Glx ratio in EZ: 1.7:1</td><td>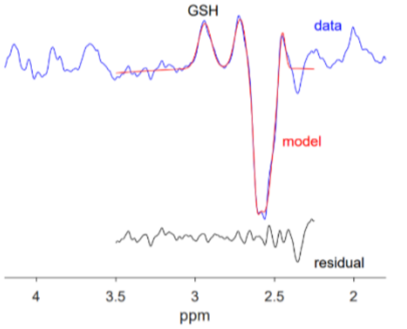
GSH/Cr: 0.04</td></tr>
<tr><td>After real tDCS</td><td>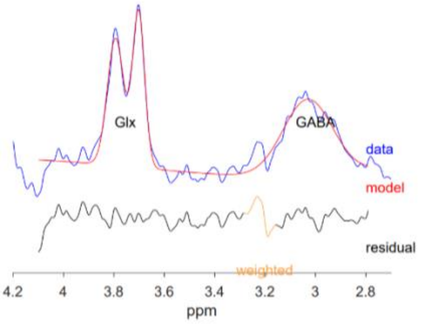
GABA/Cr: 0.10
Glx/Cr: 0.10
GABA/Glx ratio in EZ: 1:1</td><td>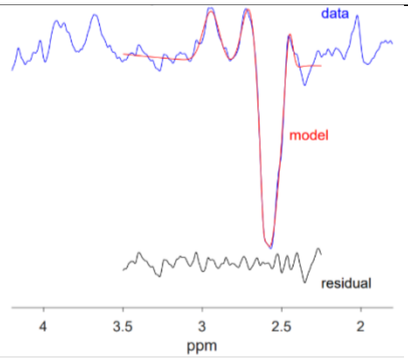
GSH/Cr: 0.05</td></tr>
</table>
